# Probe versus microscope: a comparison of different methods for image-to-patient registration

**DOI:** 10.1007/s11548-018-1800-0

**Published:** 2018-06-05

**Authors:** Martina Perwög, Zoltan Bardosi, Georgi Diakov, Olivia Jeleff, Florian Kral, Wolfgang Freysinger

**Affiliations:** 1 ENT/4D-Visualization, Medical University Innsbruck, Anichstr. 35, Innsbruck, Austria; 2 ENT, Klinikum der Universität München, Munich, Germany; 3 ENT, Kardinal Schwarzenberg Klinikum, Schwarzach im Pongau, Austria

**Keywords:** Registration, Microscope, Navigation, Target registration error

## Abstract

**Purpose:**

Computer-aided navigation is widely used in ENT surgery. The position of a surgical instrument is shown in the CT/MR images of the patient and can thus be a good support for the surgeon. The accuracy is highly dependent on the registration done prior to surgery. A microscope and a probe can both be used for registration and navigation, depending on the surgical intervention. A navigation system typically only reports the fiducial registration error after paired-point registration. However, the target registration error (TRE)—a measurement for the accuracy in the surgical area—is much more relevant. The aim of this work was to compare the performance of a microscope relative to a conventional probe-based approach with different registration methods.

**Methods:**

In this study, optical tracking was used to register a plastic skull to its preoperative CT images with paired-point registration. Anatomical landmarks and skin-affixed markers were used as fiducials and targets. With both microscope and probe, four different registration methods were evaluated based on their TREs at 10 targets. For half of the experiments, a surface registration and/or external fiducials were used additionally to paired-point registration to study their influence to accuracy.

**Results:**

Overall, probe registration leads to a smaller TRE ($1.69 \pm 0.74\,\hbox {mm}$) than registration with a microscope ($2.19 \pm 0.94\,\hbox {mm}$). Additional surface registration does not result in better accuracy of navigation for microscope and probe. The lowest mean TRE for both pointers can be achieved with paired-point registration only and radiolucent markers.

**Conclusion:**

Our experiments showed that a probe used for registration and navigation achieves lower TREs compared using a microscope. Neither additional surface registration nor additional fiducials on an external reference element are necessary for improved accuracy of navigated ENT surgery on a plastic skull.

## Introduction

Clinical navigation systems are commonly used for ENT interventions. At critical regions, they are supporting the surgeon by realizing the exact positions of a pointer inside the patients’ body on the CT/MR images. Depending on the surgical intervention, different pointers can be used for navigation; for example, a microscope is required at the lateral skull base to support the surgeon with detailed images of the surgical area. In this case, the pointer can be the microscope or a probe.

**Table 1:** Overview of the different experiments with the number of fiducials and targets used for registrations and measurements

Pointer		# Fiducials					# Targets		
Probe	Microscope	Anat.	Donut	Radiolucent	Surface reg.	VBH	Anat.	Donut	Radiolucent
A1	B1	5	2	3	–	–	5	3	2
A2	B2	5	2	3	25	–			
A3	B3	3	1	2	–	4			
A4	B4	3	1	2	25	4			

Prior to using a navigation system, the CT/MR images have to be registered to the patient. In general, the two main registration methods are paired-point and surface registration [1, 2]. For the first method, points on the patient (‘fiducials’) have to be located, together with the corresponding points in the images. Thus, the rigid transformation between image and patient coordinates can be calculated. Besides, anatomical structures also skin-affixed or bone-implanted markers can be used as fiducials. Skin-affixed markers are known to be prone to changes of the skin according to different hydration statuses or effects of muscle relaxing agents and thus are to be used with caution [3]. Those aspects can be avoided if bone-implanted fiducials are used, but because of the invasiveness they are not the first choice, due to side effects for the patient. The most commonly used fiducials are anatomical landmarks, as they are easy to use and non-invasive.

Surface registration helps automation and registers a larger number of points to the image. These points are collected by moving a pointer over a large surface on the patient, like the forehead. With the iterative-closest-point (ICP) algorithm, the surface of the patient is matched to the corresponding 3D-image-model [2].

For interventions, e.g. the lateral skull base or the petrous bone, the VBH (Vogele–Bale–Hohner) head holder can be used additionally to fix the patients head on the OR Table [4]. External fiducials can be affixed to the VBH mouthpiece to get a larger area of fiducials that surrounds the surgical region and thus improve accuracy [5, 6]. More details are given in the ‘Materials and methods’ section.

The ideal case for the surgeon would of course be perfect (error free) navigation, but the registration is a critical step and has to be done as accurately as possible. However, it always introduces errors as calibration, tracking and measurement errors can occur, but user localization errors are also common in surgical navigation [7–10].

After registration, the typical navigation systems report a single error which is called the fiducial registration error (FRE) [11]. The FRE is the RMS distance of CT image fiducials and patient fiducials that are transformed to the CT image. However, the target registration error (TRE) is much more important for the surgeon in practice. It reports the accuracy at a target location close to the surgically relevant positions and is uncorrelated with the FRE [12].

Unfortunately, not many studies regarding the application accuracy (the TRE) of different registration methods have been done comparing microscope and probe registration methods [13–19].

Therefore, the aim of this study was to find the optimal registration method of a plastic skull to its preoperative CT images in terms of application accuracy at the lateral skull base, concerning the pointer and the registration method. Two different questions were evaluated: What kind of pointer should be used for registration and measurements? Which registration method is the optimal method to register a plastic skull to its CT images?

Two different pointers were used for both registration and TRE measurement: a microscope and a pointer. Both pointers are optically tracked by a camera relative to a DRF (digital reference frame), so the coordinates of the points in patient space can be determined and transformed to image space.

Four different registration methods have been experimentally evaluated with each pointer (probe or microscope), two of them with external fiducials on the VBH mouthpiece. Surface registration was additionally used for half of the experiments (see Table 1 for a detailed description). The TRE was measured on 10 different targets, and all methods were compared to each other. Thus, it was possible to evaluate the performance and differences of the registration methods and pointers used for our experiments.

## Materials and methods

**Fig. 1:**
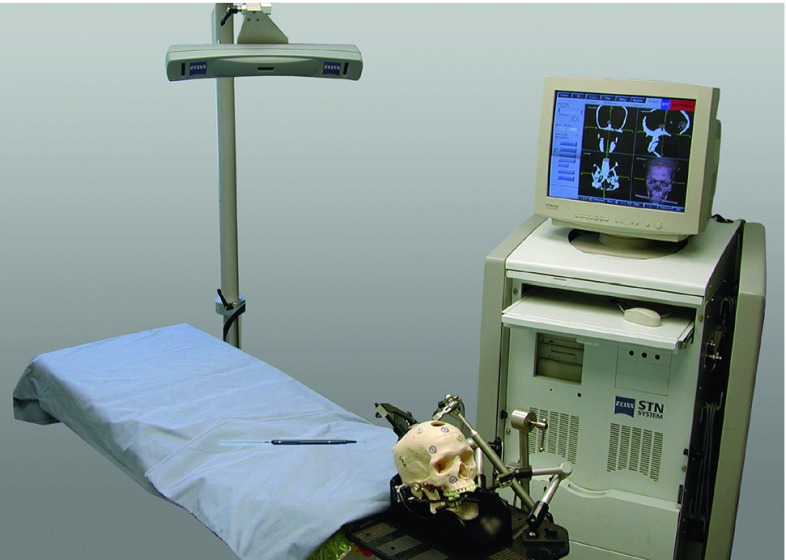
Experimental set-up for experiments A1,..., A4. The plastic skull is fixed with the VBH head holder on the OR table. The camera is optimally placed and the navigation system placed opposite to the surgeon [23]

**Fig. 2:**
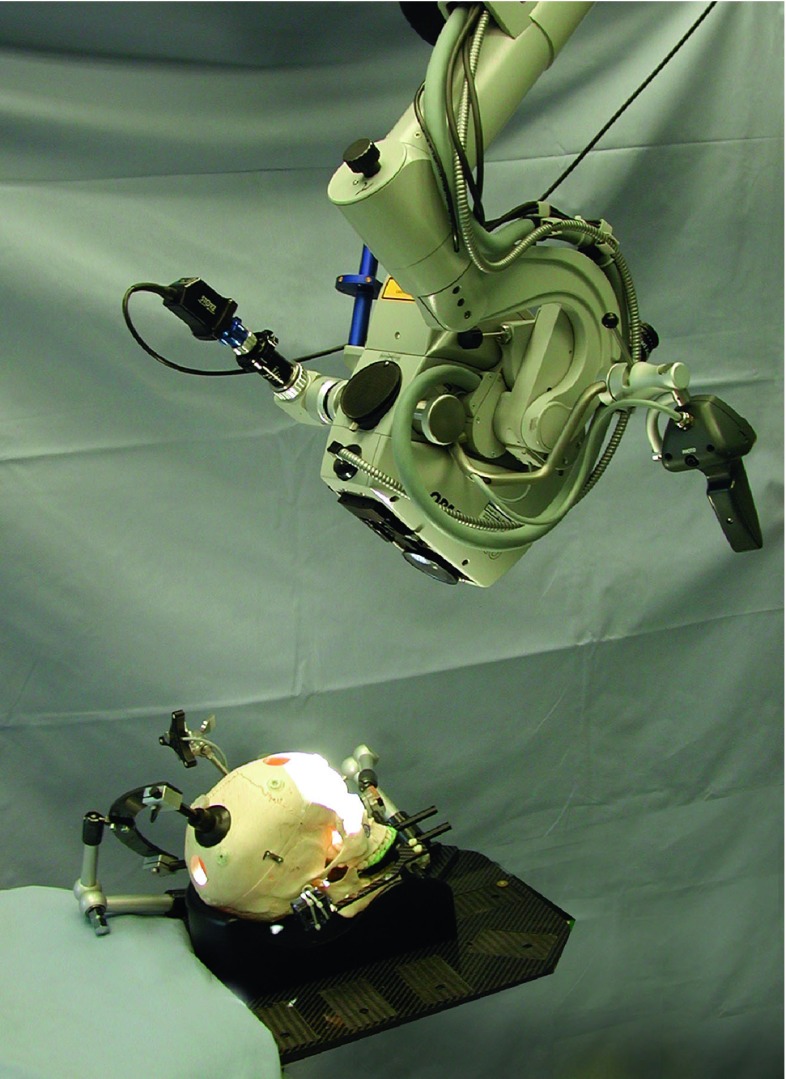
Experimental set-up when the microscope is used for the registration. The DRF is fixed on the left side of the microscope (the blue cylinder hidden in this figure on the left side) [23]

The experimental set-up can be seen in Figs. 1, 2.

### Phantom

**Fig. 3:**
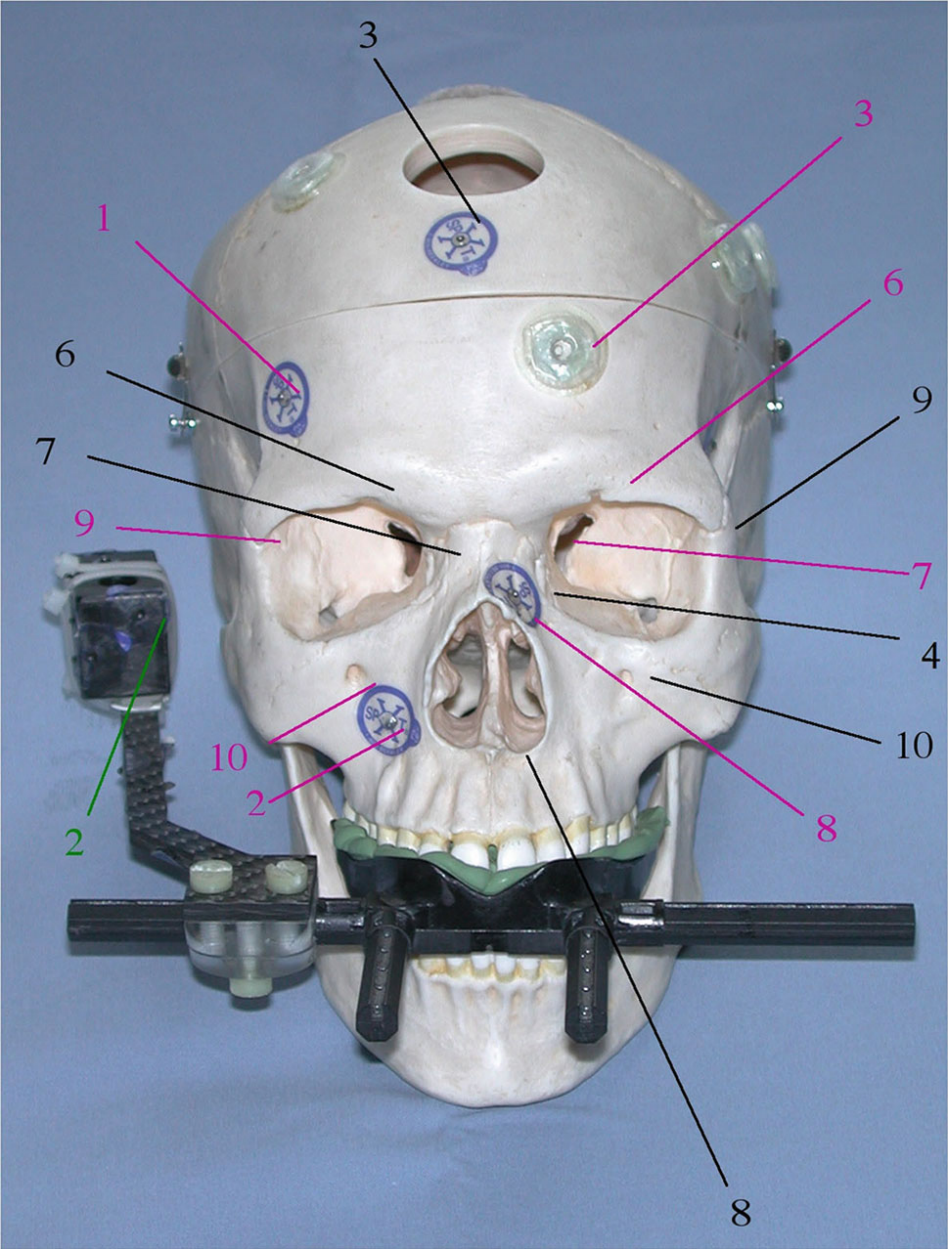
Plastic skull with the VBH mouthpiece. Targets are shown in pink; fiducials are shown in black. The number in green shows the fiducial used for registration only with VBH mouthpiece. (fiducials: 3—X-spot on the frontal bone, 4—X-spot on the left nasal bone, 6—anatomical landmark on the right supraorbital foramen, 7—right inner cantus, 8—anterior nasal spine, 9—saddle point on the left frontozygomatic suture, 10—infraorbital foramen; targets: 1, 2—radiolucent markers on the right frontal bone and the right maxillary bone, 3—donut marker on the left frontal bone, 6—left supraorbital foramen, 7—left inner canthus, 8—left nasal bone, 9—saddle point right frontozygomatic suture, 10—right infraorbital foramen) [23]

**Fig. 4:**
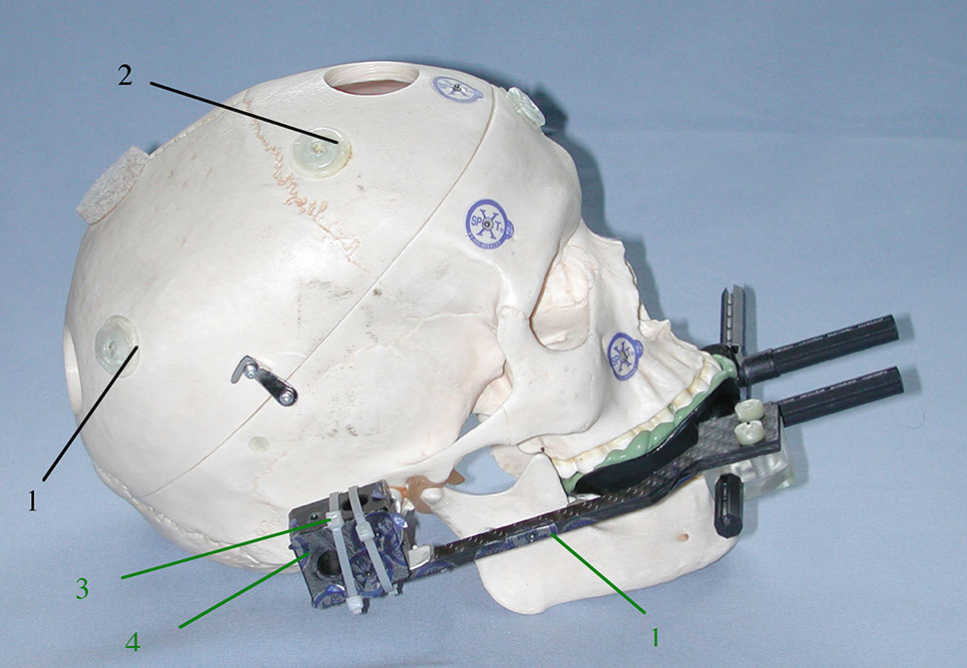
Plastic skull from the right with fiducials (black) and fiducials on the VBH mouthpiece (green). (1—Donut marker on the right parietal bone, 2—Donut marker on the right frontal bone, 3 and 4—X-spots on the VBH—mouthpiece used only for registration with the VBH mouthpiece) [23]

A plastic skull (Somso, Coburg, Germany) was used for all experiments. Radiolucent markers (X-spot, izi-Kooperation, Beekley Corp., Conn, USA) and MR marker (Donuts, izi-Kooperation, Beekley Corp., Conn., USA) were attached on the skull. For all experiments the plastic skull was fixed on an OR table with a VBH head holder [4]. With the VBH mouthpiece, the skull can be attached at the holder [6]. Dental foam vacuum fixation on the maxilla leads to a stable construction in all experiments. This is a fast and secure non-invasive method to fix a patient during interventions. On the reference element of the VBH mouthpiece, radiolucent markers were attached (see Figs. 3, 4). Those external markers were only used for half of the experiments (see Table 1).

### CT images

CT images of the plastic skull were acquired with a Siemens-Sensation-16 (Siemens, Erlangen, Germany), leading to 2 axial datasets. The first dataset was captured with the reference element fixed on the VBH mouthpiece in place and the second one without the reference element. Image parameters were 140 kV, 220 mAs with 1 mm slice thickness.

### Navigation system

The SNN navigation system (Fa. SNS, Aalen, Germany) was used for navigation with optical tracking of (flashpoint-3000-system, IGT, Boulder, USA). It comprises classical pointer-based navigation and a navigated microscope with heads-up display capabilities for navigation.

### Pointers

Two different pointers were used for registration and navigation on a plastic skull. The probe (135-mm flashpoint) can be optically tracked by the camera with the two LEDs attached to it and is connected with a cable to the navigation system.

The microscope (OPMI 2000, Zeiss, Oberkochen, Germany) can be optically tracked with a DRF attached to it (see Fig. 2). The microscope is factory-calibrated, and thus, the parameters of the camera are known and the position of the point that is in focus can be determined.

### Registration

To register the plastic skull to its CT images, four different registration methods were performed with the probe (group A) or the microscope (group B). As a skull has only a few prominent anatomical landmarks that can be defined well both on the patient and in the CT images, different kinds of markers were used as fiducials (but also targets, as can be seen in the next section), to have a sufficient amount of fiducials for each registration method.

The different methods and fiducials used for registration were defined as follows (see also Table 1):

A1 and B1: paired-point registration with 5 anatomical landmarks, 2 donut markers and 3 radiolucent markers

A2 and B2: paired-point registration with 5 anatomical landmarks, 2 donut markers, 3 radiolucent markers and additionally a surface registration with 25 points

A3 and B3: paired-point registration with 3 anatomical landmarks, 1 donut marker and 2 radiolucent markers on the skull and 4 radiolucent markers on the VBH mouthpiece

A4 and B4: paired-point registration with 3 anatomical landmarks, 1 donut marker and 2 radiolucent markers on the skull, 4 radiolucent markers on the VBH mouthpiece and additionally a surface registration with 25 points

The probe and the microscope were both used as pointers to locate a fiducial on the plastic skull. The fiducials were physically located with the tip of the probe. With the microscope, the fiducials were located with maximum magnification. Only when the fiducial of interest was in autofocus and the microscope was not moving anymore, this point was used for registration.

**Fig. 5:**
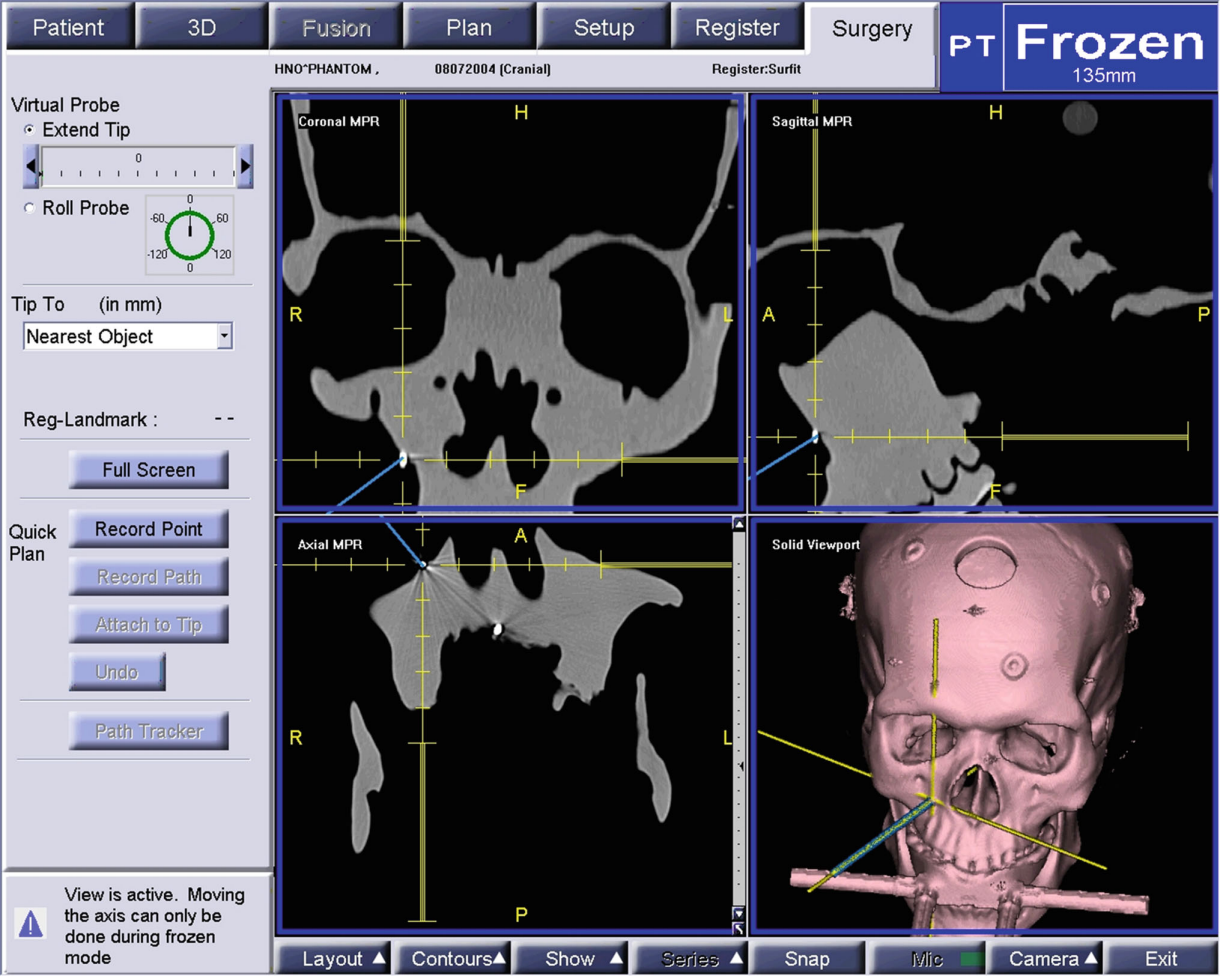
Screenshot of the navigation system’s monitor when pointing on the X-spot. The difference between the target point and the pointer tip is measured in axial, coronal and sagittal views (c.f. Fig. 6, right). The crosshair is used to get the scaling for the measurement (c.f. Fig. 6, left) [23]

**Fig. 6:**
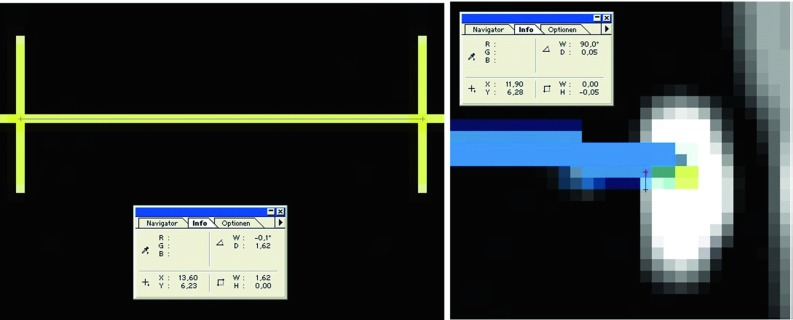
Left side: measuring the length of the crosshair, which is defined as 1 cm. The measured value is 1.62 in this image and is used as the scaling factor for the measurements. Right side: example of the TRE measurement on a X-spot. Maximized part of the image of the display of the navigation system. The probe (blue) is pointing at the target (white elliptical point). The difference between the tip of the probe and the centre of the target is 0.05 [23]

In detail (cf. Figs. 3, 4), the fiducials used for the experiments *without* VBH mouthpiece are the 2 donut markers on the right parietal bone and the right frontal bone, the 3 radiolucent markers on the frontal bone, the left nasal bone and the left temporal bone. The five anatomical landmarks are the right supraorbital foramen, the right medial cantus, the anterior nasal spine, the ‘saddle point’ on the left frontozygomatic suture, and the left infraorbital foramen.

For the experiments *with* the VBH mouthpiece, the fiducials in detail are 4 radiolucent markers on the referencing element of the VBH mouthpiece, 1 donut marker at the right parietal bone, 2 radiolucent markers median on the frontal bone and the left temporal bone and the 3 anatomical landmarks on the anterior nasal spine, the left infraorbital foramen and the right supraorbital foramen.

For the surface registration, 25 points were acquired by the probe touching the mid-face, the frontal bone and the orbitae of the skull surface. The same anatomical locations were focused with the microscope with maximum magnification. Again 25 points were selected, when the points were in focus and the microscope was not moving.

### TRE measurements

After all different registration methods, the TRE was measured on 10 targets, which were per definition different to the fiducials (c.f. Fig. 3). The targets used were always the same for A1–A4 and B1–B4.

Five anatomical landmarks, 2 radiolucent markers and 3 donut markers were used. In detail, the targets (in ascending order target number 1–10) are the 2 radiolucent markers on the right frontal bone and the right cheek bone, the donut markers on the left frontal bone, near the left coronal suture and the left parietal bone. The 5 anatomical targets are the left supraorbital foramen, the left medial cantus, the bony structure on the left nasal bone, the ‘saddle point’ on the right frontozygomatic suture and the right infraorbital foramen.

Each experiment (registration and TRE measurement) was repeated 10 times. To avoid a learning effect during the experiments, initial experiments were done to get used to the equipment without recording the results.

Each target was located as exactly as possible with the probe. A screenshot of the navigation systems display was taken, when the probe was placed at the target. The same procedure was repeated with the microscope, and each target was located with the maximum magnification and with autofocus.

After finishing the experiments, each screenshot was processed in Adobe Photoshop 5.0. Each image plane was maximally magnified. The difference of real target and projected target ($\varDelta {x}$, $\varDelta {y}$, $\varDelta {z}$) was measured in each image plane, axial (*x*–*y*), sagittal (*x*–*z*) and coronal (*y*–*z*). So each *x*-, *y*- and *z* direction was measured twice (10 $\times$ 6 data matrix for each group). Those pixel measurements were converted in mm. The scale is known by measuring the length of the crosshair, which is 1 cm. An example of the TRE measurement is shown in Figs. 5, 6.

It could be possible that the target was not visible in one plane, and thus, the error could only be measured in the other two planes. The missing values were assigned as NaN (not-a-number) and replaced with the corresponding value in *x*-, *y*- and *z*-directions; for example, if the axial *x*-direction could not be measured, the measurement of the sagittal *x*- direction was used for both *x*-values.

Overall 4800 measurements were taken, 600 for each experiment. For each target, the mean of the 10 repetitions in each direction was calculated. Thus, the values ($\Delta x_{{\textit{ij}}}$, $\Delta y_{{\textit{ij}}}$, $\Delta z_{{\textit{ij}}}$) remained for each target. With these values, the TRE was calculated by

$$\begin{aligned} \mathrm{{TRE}} ({t_{{\textit{ij}}}}) = \sqrt{\Delta x_{{\textit{ij}}}^2 + \Delta y_{{\textit{ij}}}^2 + \Delta z_{{\textit{ij}}}^2}, \end{aligned}$$

which is the TRE for target number *i*, $i = 1, {\ldots }, 10$, experiment *j*, $j = \hbox {A1},{\ldots }, \hbox {A4}, \hbox {B1},{\ldots }, \hbox {B4}$.

All calculations were done with all measurements of each group in Matlab.

Besides that, a worst-case scenario was analysed, where only the largest values in each *x*-, *y*-, and *z*-directions of each repetition were used for calculations (10 $\times$ 3 data matrix for each group).

**Table 2:** TREs with the *p* values of the Wilcoxon signed-rank test

TRE (mm)	A1	B1	A2	B2	A3	B3	A4	B4
Mean ± std	1.41 ± 0.61	1.70 ± 0.68	1.48 ± 0.61	3.02 ± 1.37	1.44 ± 0.50	1.92 ± 0.72	2.44 ± 1.09	2.10 ± 0.81
*p* value	0. 0645		0.0020		0. 0273		0.1602	

It can be seen that A2–B2 and A3–B3 are statistically not equal. ($\hbox {H}_{0}$: $\hbox {A}_{\mathrm{i}} = \hbox {B}_{\mathrm{i}}, \hbox {i} = 1, {\ldots }, 4$). Mean values and standard deviations are given in mm

### Evaluation

The aim of this investigation was to find the difference regarding the TRE between registrations via microscope and probe on a plastic skull. Besides that, it was evaluated if surface registration (additionally to paired-point registration) and additional fiducials fixed on the reference element of the VBH mouthpiece improve the TRE. Group A (probe) and group B (microscope) were compared to each other. The overall TRE over all four types of registration was calculated for a first overview, but also the TRE of each registration method was calculated separately. All calculations were done with Matlab R2012a. Statistical tests were done with $\alpha = 0.05$. An ANOVA was used to find differences in group A or group B. If a statistically significant difference was found, multiple comparisons were done with Bonferroni correction to analyse which experiments were significantly different to each other. For comparing $\hbox {A}i$ and $\hbox {B}i$, $i = 1, {\ldots }, 4$, a Wilcoxon signed-rank test was used.

## Results

All results are mean values with their standard deviation and can be seen in detail in Tables 2, 3. Group A defines the registration with the probe and group B the registration with the microscope.

**Table 3:** Mean TRE at different landmarks used as targets for the 8 groups

TRE (mm)	A1	B1	A2	B2	A3	B3	A4	B4
Radiolucent X-marker	0.94 ± 0.35	1.59 ± 0.64	0.78 ± 0.31	2.10 ± 0.97	1.25 ± 0.46	1.48 ± 0.64	1.68 ± 0.45	1.31 ± 0.49
Donut marker	1.38 ± 0.68	1.69 ± 0.73	1.80 ± 0.85	4.21 ± 1.94	1.43 ± 0.56	2.20 ± 0.85	2.74 ± 1.19	2.77 ± 1.18
Anatomical landmarks	1.62 ± 0.65	1.77 ± 0.67	1.58 ± 0.52	2.69 ± 1.08	1.53 ± 0.49	1.94 ± 0.67	2.57 ± 1.20	2.02 ± 0.62

All errors in the groups, except A1, A2 and B2, are normally distributed (${p} < 0.05$). All 8 groups compared to each other are not equal (ANOVA, ${p} < 0.0001$); an overall difference of microscope- and probe-based registration can also be observed (Wilcoxon signed rank, ${p} < 0.0001$).

The overall TRE for group A is $1.69 \pm 0.74 \, \hbox {mm}$, and for group B, it is $2.19 \pm 0.94 \, \hbox {mm}$.

The mean TRE of A1 is 1.41 mm compared to B1 with a mean TRE of 1.70 mm. The mean TRE of A2 is 1.48 mm compared to B2 with a mean TRE of 3.02. A3 and B3 have a mean TRE of 1.44 mm and 1.92 mm, respectively. A4 and B4 have a mean TRE of 2.44 mm and 2.10 mm, respectively (Table 2).

These results are leading to statistical results as follows: A1 is equal to B1 ($p = 0.0645$) and A4 is equal to B4 ($p = 0.1602$). A2 and B2 are significantly different ($p = 0.0020$), as well as A3 and B3 ($p = 0.0273$).

The four probe groups (A1–A4) are significantly different to each other ($p = 0.0004$). A4 has the largest error.

B1–B4 are significantly different to each other ($p = 0.0012$), and B2 has the largest TRE.

Regarding the error in *x*-, *y*- and *z*-directions, it could be observed that overall the TRE is anisotropic. For the probe measurements, the error in *y*-direction is the smallest with $0.79 \pm 0.49 \, \hbox {mm}$ and in *x*- and *z*-directions the error is $1.00 \pm 0.56 \, \hbox {mm}$ and $1.03 \pm 0.62 \, \hbox {mm}$, respectively. This is also valid for the microscope; in *y*-direction TRE is $1.08\pm 0.60 \, \hbox {mm}$, in *x*-direction $1.31 \pm 0.80 \, \hbox {mm}$ and in *z* direction $1.27 \pm 0.72\,\hbox {mm}$.

The TRE of anatomical targets and skin-affixed markers can be seen in Table 3. Skin-affixed markers have a TRE of $1.50 \pm 0.67 \, \hbox {mm}$ for group A and $2.16 \pm 1.02 \, \hbox {mm}$ for group B. The TRE for the anatomical landmarks is $1.82 \pm 0.77 \, \hbox {mm}$ (group A) and $2.10 \pm 0.78 \, \hbox {mm}$ (group B).

**Table 4:** Worst-case scenario. TREs with the *p* values of the Wilcoxon signed-rank test

TRE (mm)	A1	B1	A2	B2	A3	B3	A4	B4
Mean ± std (worst case)	1.69 ± 0.63	1.93 ± 0.71	1.83 ± 0.68	3.37 ± 1.46	1.68 ± 0.52	2.22 ± 0.74	2.74 ± 1.19	2.43 ± 0.85
*p* value	0.1934		0.0020		0.0195		0.4315	

It can be seen that A2–B2 and A3–B3 are statistically not equal ($\hbox {H}_{0}: \hbox {A}_{\mathrm{i}} = \hbox {B}_{\mathrm{i}}, \hbox {i} = 1, {\ldots }, 4$). Mean values and standard deviations are given in mm

Dividing skin-affixed markers in radiolucent and donut markers, the TRE of group A is $1.16 \pm 0.40$ and $1.84 \pm 0.85 \, \hbox {mm}$, respectively, and of group B $1.62 \pm 0.71\,\hbox {mm}$ and $2.71 \pm 1.27\,\hbox {mm}$, respectively.

**Fig. 7:**
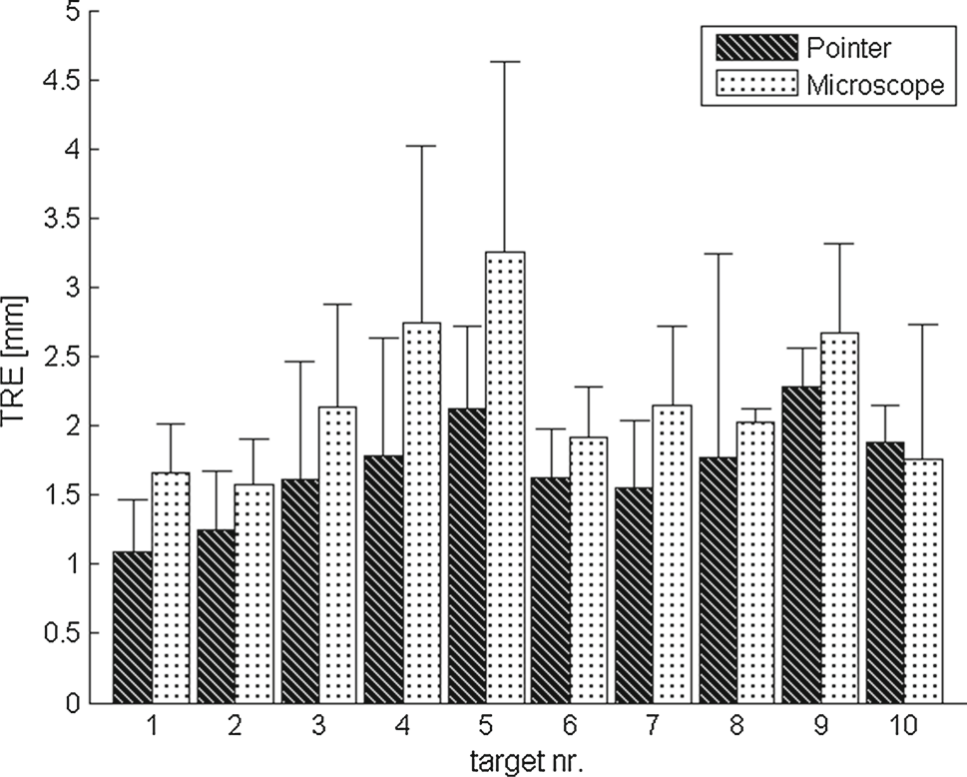
Pointwise TRE (in mm). On each target, the TRE was calculated over all registration methods. The black bar is the mean TRE of target 1–10 when the probe is used (A1–A4), the white bar is the mean TRE for target 1–10 when the microscope is used (B1–B4). Targets 1–10 are shown and described in Fig. 3

Pointwise evaluation is shown in Fig. 7. Target number 10 (right infraorbital foramen) is the only point, where using the probe leads to a larger TRE than using the microscope.

The results of the worst-case scenario can be seen in Tables 4, 5. The TRE for group A is $1.98 \pm 0.80\,\hbox {mm}$ which means a deterioration of the mean of 0.29 mm in comparison with the overall result. For group B, the worst-case TRE is $2.49 \pm 0.99 \, \hbox {mm}$; this is a deterioration of the mean of 0.30 mm compared to the overall TRE.

**Table 5:** Worst-case scenario: Mean TRE for the different landmarks used as targets for all registration methods

TRE (mm) Mean ± std	A1	B1	A2	B2	A3	B3	A4	B4
Radiolucent X-marker	1.05 ± 0.36	1.67 ± 0.67	0.89 ± 0.33	2.25 ± 1.01	1.45 ± 0.46	1.61 ± 0.65	1.77 ± 0.47	1.45 ± 0.49
Donut marker	1.75 ± 0.69	1.94 ± 0.78	2.36 ± 1.00	4.66 ± 2.06	1.78 ± 0.56	2.55 ± 0.91	3.13 ± 1.26	3.26 ± 1.26
Anatomical landmarks	1.90 ± 0.68	2.03 ± 0.68	1.89 ± 0.53	3.05 ± 1.15	1.71 ± 0.52	2.26 ± 0.66	2.90 ± 1.33	2.32 ± 0.64

The worst-case TRE for group A and skin-affixed markers is $1.77 \pm 0.71\,\hbox {mm}$ ($1.29 \pm 0.41\,\hbox {mm}$ radiolucent markers and $2.26 \pm 0.92 \, \hbox {mm}$ donut markers) and $2.10 \pm 0.83 \, \hbox {mm}$ for anatomical landmarks (Table 5).

Group B has a worst-case TRE of $2.41 \pm 0.81\,\hbox {mm}$ for the anatomical landmarks and $2.42 \pm 1.09\,\hbox {mm}$ ($1.75 \pm 0.73$ radiolucent markers and $3.10 \pm 1.35\,\hbox {mm}$ donut markers).

For the worst-case scenario, no significant changes occurred to the statistical results and only the *p* value for A4–B4 increased to 0.43.

## Discussion

This study evaluated four different registration methods using two different pointers. From a first point of view, it was apparent that the microscope led to larger TREs than the probe. However, with additional surface registration plus using the VBH mouthpiece, the probe registration led to larger TREs than the microscope. Statistically no difference between microscope and probe registration could be found in this case. In general, more errors are influencing the accuracy of the microscope compared to the probe. Both pointers are tracked, but the error chain of the microscope also includes camera calibration transformation errors. From this aspect, it can also be argued that the accuracy of the microscope is similar to that of the probe.

In general, surface registration is more time intense than paired-point registration. But also locating the fiducials and targets with the microscope takes much more time due to difficulties in focusing the points. Those two facts were already speaking against using the microscope and an additional surface registration before any calculations were done. However, it is evident that some interventions, e.g. at the petrous bone, cannot be done without a navigated microscope and thus the time factor is negligible.

The worst results can be observed for B2 and A4, i.e. paired-point combined with surface registration is less accurate/precise than paired-point registration only. This result was also found in [14].

However, there was no (statistically significant) difference between A1, A2 and A3. All those methods led to a mean TRE lower than 1.5 mm. Though it has to be said that A1 was the method that needed the fewest preparations and was ‘easy to use’. Thus, paired-point registration only is an adequate method for our experiments.

Regarding the registration with the microscope, B1, B3 and B4 were statistically equal. Using the microscope was more time-consuming due to the waiting time until the microscope is at rest and the autofocus is adjusted to confirm a fiducial/target. However, when only paired-point registration was applied, the time factor can be neglected and the TRE was similar to the experiments done with the probe.

Pointwise evaluation clearly showed that donut markers were the worst targets to use. This may be caused by the form of this marker, which is difficult to focus with the microscope. It is also possible that those markers were difficult to locate in the CT images, or the centre might not have been clearly visible. For our experiments, it would have been better not to use donut markers as a target. Anatomical landmarks showed better TREs, the smallest values could be obtained at X-spots. The targets with the largest mean TRE when using the probe were the anatomical marker target nr. 9, and the Donut marker target nr. 5 when the microscope was used.

It is clear that on a plastic skull anatomical landmarks and skin affixed markers can be located more accurately in contrast to a real patient, where the skin is moving, or bony structures, like foramina cannot be touched precisely. Besides that, it is hard to realize not using anatomical landmarks, because they are easy to use, non-invasive and clinical standard.

Anisotropy of the TRE in *x*-, *y*- and *z*-directions could be detected. In our experiments, the mean TRE is about 0.3 mm smaller in *y*-direction than in *x*- and *z*-directions. Typically, in optical systems the viewing direction has the largest noise variance, e.g. [20]. The anisotropy found in our set-up can be caused by not orienting the probe/microscope correctly to the camera, the underlying FLE, or the variance of the camera. Besides that, the slice thickness of the CT images is larger than the pixel resolution of the images.

The effect of the location of the fiducials that are used for registration is an interesting point which can influence the accuracy of navigation. The fiducials for paired-point registration are the same for A1/B1 and A2/B2, but also for A3/B3 and A4/B4. As shown in the results only A4 and B2 are significantly different to the other groups. But both methods are using surface registration in addition that should improve results, especially as a rigid surface was used [21]. The standard deviations for both A4 and B2 are about the double of the standard deviations of A1, A2, A3 and B1, B3, B4, respectively. Registration, measurement or user errors could be the cause for this discrepancy. Besides that, the difference seems to be caused due to difficulties in locating the targets. Thus, it seems that external fiducials on the VBH mouthpiece do influence the accuracy of navigation in a negative way when a microscope is used.

**Fig. 8:**
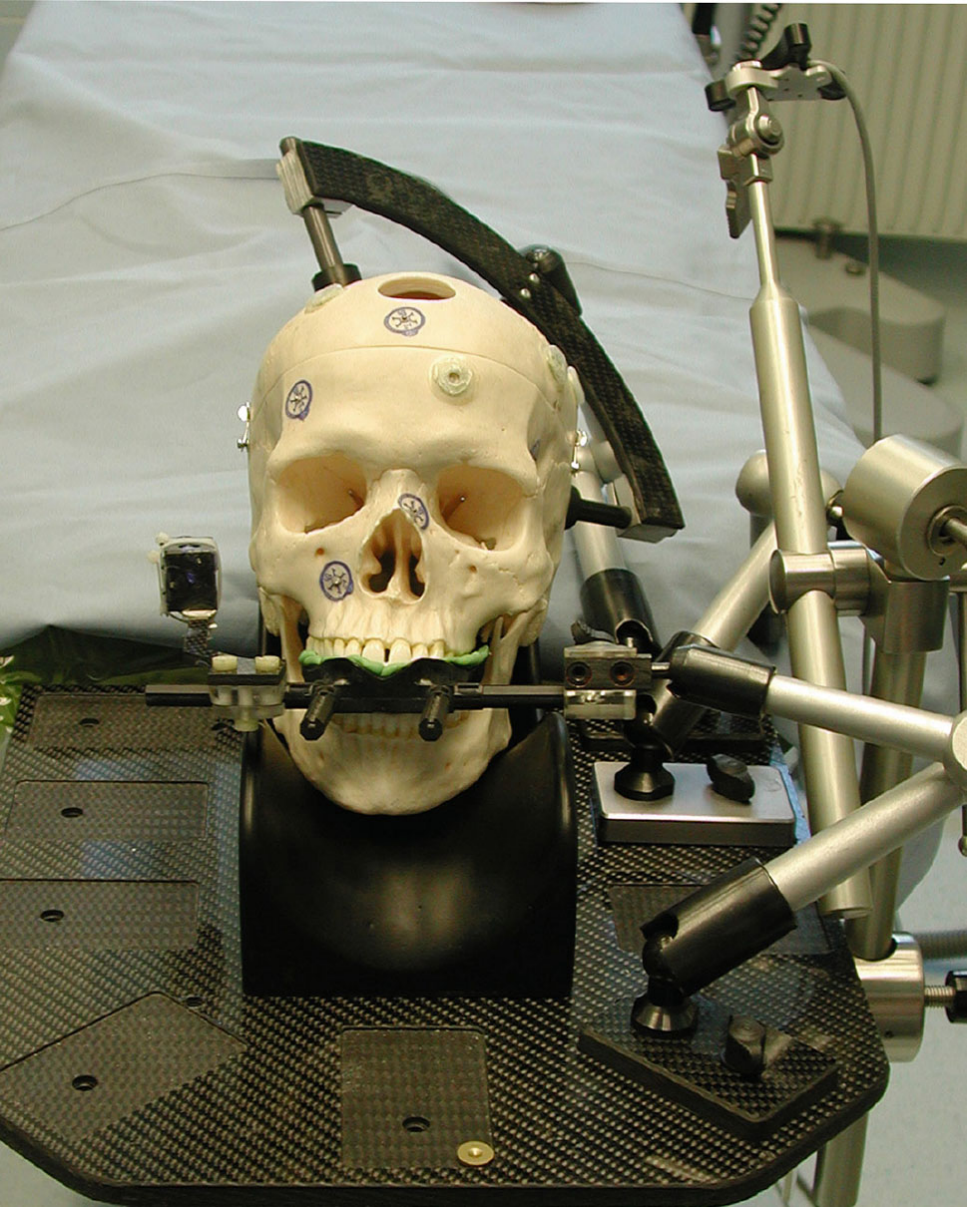
Plastic skull with the VBH mouthpiece fixed with the VBH holder to the OR table. The DRF can be seen on the right side of the plastic skull [23]

Analysing the worst-case scenario, it can be observed that the deterioration of the mean TRE is in all cases not more than 0.35 mm, the standard deviation staying quite constant for all cases. This means that measurements were good and constant, without large outliers. Considering the different types of targets, the values of the X-spots increase about 0.1 mm, but Donut markers have mean values more than 0.5 mm worse. Anatomical landmarks have a mean TRE that is about 0.4 mm worse than in the overall case. This speaks again against using Donut markers but also against anatomical landmarks, because locating them is difficult and leads to a large error. In contrary to the overall scenario, target 5 has the largest mean TRE for both groups A and B in the worst-case scenario. This target is a Donut marker on the left parietal bone.

Overall the largest TREs can be observed on the targets 5 and 9 for both groups (Donut marker on the left parietal bone and the saddle point on the right frontozygomatic suture), the smallest TREs on targets 1 and 2 (X-spots). Due to the experimental setting, locating target 5 might be difficult (Fig. 8). Target 9 is a very unspecific point and is difficult to locate both in the CT image and on the plastic skull, in contrary to targets 1 and 2, which are X-spots, that are easy to locate on the front of the skull. X-spots can be located more precisely than all other markers, they have the smallest standard deviations. In contrary to that, Donut markers have the largest standard deviations; only in experiments A4 and B4, anatomical landmarks can be located less precisely.

Unfortunately, the study is limited to a paired-point registration using 10 fiducials throughout the experiments. As already confirmed by other studies (e.g. see [8, 10, 22]), a smaller number of fiducials lead to a larger TRE than using more fiducials for registration. However, using, for example, 5 fiducials is not necessarily leading to a larger TRE than using 10 fiducials. It is always depending on the situation and on the fiducials used. Due to not using a different number of fiducials for the same experiment, we do not know if this would lead to an improvement for one of the registration methods. However, the 10 fiducials used were placed ideally on the plastic skull to ensure a good configuration for the registration process.

## Conclusion

The target registration error of different registration pointers and methods was evaluated, and differences between the alternative approaches could be observed. Overall it can be observed that a probe used with paired-point registration gives the best results for navigated surgery. Additional surface registration and/or fiducials do not improve the TRE significantly in our experiments. Using a microscope as a pointer leads to a larger error compared to the probe for all experiments except when paired-point registration is combined with external fiducials and surface registration. However, in clinical practice pointers are mostly used for registering the patient to pre- and intraoperative radiological data.

In conclusion, both registration instruments had comparable application accuracies for the plastic skull in an experimental set-up. Using the microscope is a rarely used registration device, but this study demonstrates that registration outcome is not affected by the registration tool chosen.
